# Role of Brush Biopsy and DNA Cytometry for Prevention, Diagnosis, Therapy, and Followup Care of Oral Cancer

**DOI:** 10.1155/2011/875959

**Published:** 2010-12-20

**Authors:** Alfred Böcking, Christoph Sproll, Nikolas Stöcklein, Christian Naujoks, Rita Depprich, Norbert R. Kübler, Jörg Handschel

**Affiliations:** ^1^Institute of Cytopathology, Heinrich-Heine-University, Moorenstra*β*e 5, 40225 Düsseldorf, Germany; ^2^Department for Cranio- and Maxillofacial Surgery, Heinrich-Heine-University, Moorenstra*β*e 5, 40225 Düsseldorf, Germany; ^3^Department of General, Visceral, and Pediatric Surgery, University Hospital Düsseldorf, Heinrich-Heine-University, 40225 Düsseldorf, Germany

## Abstract

Late diagnosis resulting in late treatment and locoregional failure after surgery are the main causes of death in patients with oral squamous cell carcinomas (SCCs). Actually, exfoliative cytology is increasingly used for early detection of oral cancer and has been the subject of intense research over the last five years. Significant advances have been made both in relation to screening and evaluation of precursor lesions. As this noninvasive procedure is well tolerated by patients, more lesions may be screened and thus more oral cancers may be found in early, curable stages. Moreover, the additional use of DNA image cytometry is a reasonable tool for the assessment of the resection margins of SCC. DNA image cytometry could help to find the appropriate treatment option for the patients. Finally, diagnostic DNA image cytometry is an accurate method and has internationally been standardized. In conclusion, DNA image cytometry has increasing impact on the prevention, diagnostic, and therapeutical considerations in head and neck SCC.

## 1. Introduction

Patients with squamous cell carcinomas of the oral cavity have a fair prognosis with an overall 5-year survival rate of about 45% [1]. Unfortunately, this figure has not substantially improved during the past 30 years [2]. Late diagnosis resulting in late treatment and locoregional failure after surgery or even after combined surgery and radiotherapy are the main causes of death in patients with oral squamous cell carcinomas.

These days, an alternative method for the examination of oral lesions is exfoliative cytology. It is based on the technique of Papanicolaou, which is accepted worldwide, as a successful method in order to screen for epithelial dysplasias in situ or invasive carcinomas of the uteri cervix. Currently, exfoliative cytology is increasingly used for early detection of oral cancer and has been the subject of intense research over the last five years [3, 4]. Significant advances have been made both in relation to screening and in the evaluation of precursor lesions [5–11]. Although mucosal biopsy is still regarded as the gold standard for definitive oral cancer diagnosis, exfoliative cytology is a valuable tool for the noninvasive evaluation of a range of potentially preneoplastic oral mucosa lesions, like leuko-/erythroplakias and lichen ruber. The cytometric detection of DNA aneuploidy in exfoliated suspicious respectively dysplastic cells, qualifies these as malignant, up to two years earlier than cytology or histology alone [12, 13].

## 2. Prevention

### 2.1. Precursor Lesions of Oral Cancer

Oral carcinogenesis proceeds through a stepwise accumulation of (cyto)genetic changes over time. Because the oral cavity is easy to examine and risk factors for oral cancer are known, there is great opportunity to improve patient outcomes through diagnosis and treatment of premalignant lesions before the development of invasive oral carcinoma [14]. In contrast to the oral premalignant *conditions*, oral premalignant *lesions* are morphologically abnormal solitary or multiple areas of mucosa that are typically white, red, speckled or verrucous in appearance. The WHO classification [15] combines leukoplakia and erythroplakia into “precursor lesions,” with a 6.8% estimated rate of transformation of oral leukoplakias to cancer. It identifies proliferative verrucous leukoplakia as a separate high risk lesion with minimal cytological atypia. Oral lichen planus, a chronic inflammatory condition, also is associated with an increased risk of cancer development of about 3% [16, 17].

### 2.2. Indications for Brush Biopsy

Screening for oral cancer and its precursor lesions may be performed by dentists, dental surgeons, and other health care professionals. Exfoliative cytology, taking brush biopsies, is advocated for evaluation of macroscopically suspicious lesions of the oral mucosa that are detected clinically by screening. This may be followed by a mucosal scalpel biopsy. Yet, exfoliative cytology may replace tissue biopsy in lesions that are clinically not obviously suspicious for malignancy but nevertheless need surveillance. As tissue biopsy is associated with lower compliance by patients as compared to brush biopsy, this noninvasive approach may lead to a higher number of investigated suspicious oral lesions and thus to an increased rate of curable cancers, identified in early stages.

## 3. Sampling of Cells

**Figure 1:**
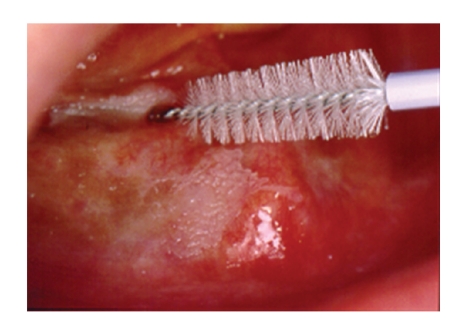
Brush biopsy from an oral verrucous leukoplakia.

Collection devices suitable to obtain cells from the superficial and intermediate layers of the oral mucosa may be conventional brushes, as used for endocervical sampling by gynecologists, such as the CytoBrush and Orca Brush (Figure 1). The brush is rotated under slight pressure several times on the suspicious lesion. Cells are then immediately smeared on glass slides and fixed with alcoholic spray. Signs of dysplasia and malignancy will also be detected cytologically in the upper layers of the squamous epithelium due to the principle of migration of cells from basal to superficial layers. The degree of nuclear abnormality in the surface layers reflects the degree of disturbance of maturation of the whole thickness of the epithelium. Thus, transepithelial sampling is not required to diagnose dysplasia and malignancy of the squamous epithelium on brush biopsies.

## 4. Assessment of Dysplasia

**Figure 2:**
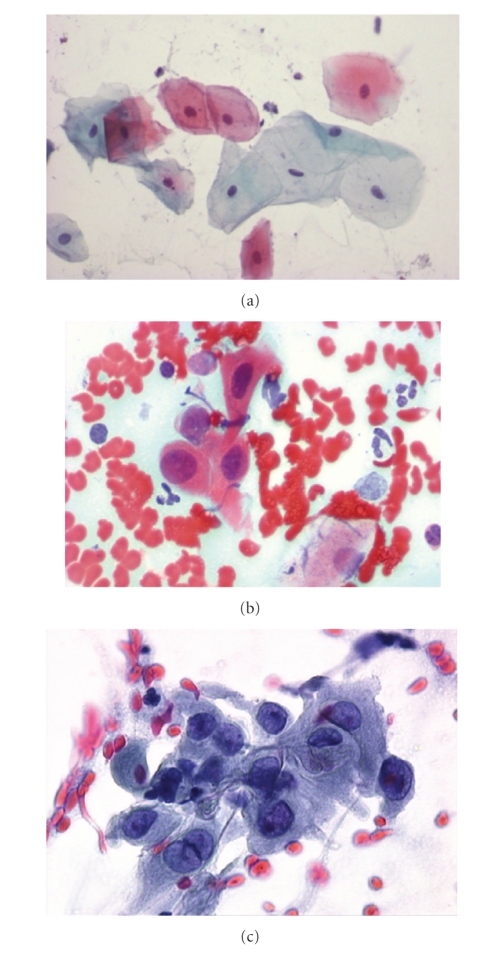
Normal (a), dysplastic (b), and malignant (c) oral squamous cells from brush biopsy, Papanicolaou stained, 630x.

There are several schemes for grading dysplasia in biopsies of oral precursor lesions. The WHO classification provides a five-step system: hyperplasia, mild, moderate, and severe dysplasia followed by carcinoma in situ [15]. Squamous cell carcinoma will develop from antecedent dysplastic oral mucosal lesions if an early diagnosis has not been made and treatment given. Early diagnosis within stages Tis or T1 correspond to a vastly improved 5-year survival rate when compared with more advanced lesions (96,7%) [17, 18]. It is the task of a cytopathologist to identify nuclear abnormalities in squamous cells collected to predict the histological grade of dysplasia. The diagnostic criteria used are well known and similar to those in cervical exfoliative cytology according to Papanicolaou [4]. Although the degree of dysplasia can be predicted on cytological samples (Figure 2), tissue biopsy is usually performed when dysplasia is detected cytologically, to confirm its grade and exclude the presence of invasion. The latter cannot be reliably assessed by exfoliative cytology alone. However, poor interobserver reproducibility in the histological assessment of oral premalignant lesions is well described [8].

## 5. Diagnostic Impact

### 5.1. Spectrum of Cytological Diagnoses

Apart from squamous cell carcinoma and its precursors (dysplasias), further neoplasias can be specifically diagnosed cytologically (e.g., naevuscell naevi, malignant melanomas, basalcell carcinomas, and malignant lymphomas). Moreover, a spectrum of non-neoplastic diseases can be differentiated using exfoliative cytology, for example, pemphigus vulgaris, Candida, herpes simplex, and HPV infections [4].

### 5.2. Diagnostic Accuracy of Cytology

Cytopathologic evaluation of oral brush biopsies from leukoplakias and erythroplakias as a single method yields sensitivities for the detection of oral cancer slightly below those of histopathologic evaluation of scalpel biopsies, reported to be 97,5% [19]. Remmerbach et al. [5, 20] documented 91,3% and 94,6% sensitivity of oral brush biopsy and Maraki et al. [12] even 100% for the detection of oral cancer, including the in situ stage. Respective specificities were 99,5%, 95,1%, and 97,4%. Moreover, 24,1% of cancers were identified in early, curable stages Tis and T1 [20]. 

It is supported by an increasing number of data that oral cytology is also a valuable technique for the assessment of oral premalignant lesions [3, 12, 21]. Exfoliative cytology has been shown to detect dysplasia in suspicious oral lesions with high sensitivity and specificity by several groups [20]. 

Up to 5–14% of oral brush biopsies may yield to equivocal cytological diagnoses [5, 20]. Underlying diagnoses are mild, moderate, or marked dysplasia, abnormal regenerating squamous epithelium, or just scarcity of abnormal cells. In these cases, ancillary methods are desirable that, nevertheless, allow more definite, correct cytological diagnoses.

Meanwhile, use of auxiliary methods such as DNA image cytometry, AgNOR analysis, and multimodal cell analysis has been shown to significantly increase diagnostic accuracy of oral cytology [12, 13, 20, 22, 23]. These methods are only applied on those samples that reveal doubtful or suspicious (dysplastic) cells, on neither cytologically normal nor frankly malignant ones.

## 6. Auxiliary Cytometry

**Figure 3:**
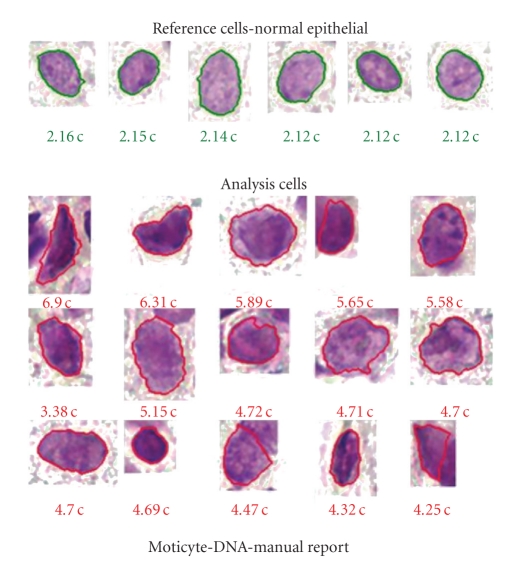
Six nuclei from normal and Feulgen-stained oral squamous cells with regular DNA content (green) as internal reference (around 2,0 c) and 15 from atypical cells with abnormal DNA content (red) between 4.25 c 6.90 c, indicative of malignancy.

**Figure 4:**
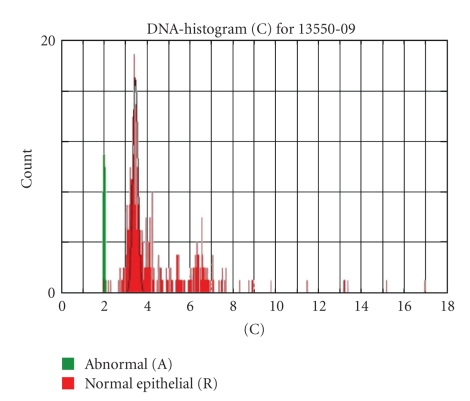
DNA histogram of in-situ oral carcinomas cells, revealing abnormal stemlines (red) at 3.5 c and 6.5 c, and values up to 17 c (DNA aneuploidy), indicative of malignancy. Normal epithelial cells (green bars) at 2 c.

DNA image cytometry is based on microdensitometric DNA measurements of several hundred atypical cells in routine cytological specimens (Figure 3). It aims to distinguish true prospectively malignant lesions (dysplasias) from microscopically atypical or otherwise doubtful ones. The biological basis of this ancillary method is chromosomal aneuploidy which is an accepted marker of malignant transformation of cells if it occurs clonally [24]. The cytometric DNA aneuploidy (Figure 4) utilizes the fact that gains or losses of chromosomes or their parts result in a plus or minus of more than 10% of nuclear DNA mass in a growing cell population (stemline aneuploidy) or if extremely high nuclear DNA values >9 c (single-cell aneuploidy) occur [24]. DNA stemlines (modal values) outside 2 c, 4 c, or 8 c ± 10% are regarded as abnormal (resp., aneuploid, Figure 4) [23, 25]. Measurements may be performed on previously stained slides after destaining and Feulgen restaining. Morphologically suspicious cells are interactively selected on a monitor, and internal calibration is performed with normal (e.g., intermediate squamous) cells (Figure 3). The method has been internationally standardized and is applicable to many different epithelial dysplasias [24–26]. After enzymatic cell separation, DNA image cytometry (ICM) can also be applied on formalin-fixed and paraffin-embedded tissues, that is on all histologic routine specimens like biopsies and resected tissues [27]. Thus, even histologic diagnoses of dysplasias can be subjected to DNA cytometry to predict their prospective behavior.

Remmerbach et al. [5] reported a frequency of 13.9% doubtful or suspicious oral cytological diagnoses due to different grades of squamous dysplasia or abnormal regenerating epithelium. Applying DNA aneuploidy as a marker for prospective malignancy on identical slides, they could improve diagnostic sensitivity of cytology for the detection of oral cancer from 91.3% to 97.8% and specificity from 95.1% to 100%. Thus 29.4% of oral cancers that clinically appeared as leukoplakias or erythroplakias were detected in stages Tis or T1. In a similar study Maraki et al. [12] described a sensitivity of 100% and specificity of 97.4% for the combined cytological and DNA cytometric evaluation of oral leukoplakias and erythroplakias. 8.1% of their cytological diagnoses had been equivocal. DNA-ICM was only applied if one of the above-mentioned diagnoses (mainly dysplasias) had occurred. Seven cases in which combined cytological/DNA cytometric diagnosis of early oral cancer was achieved up to two and half years before definitive biopsy diagnosis have been published [12, 13]. Thus DNA-ICM may help to predict the prospective behavior of cytologically suspicious lesions, as the positive predictive value of DNA aneuploid findings was reported to be 100% and the negative value 98.1% [13, 20].

**Figure 5:**
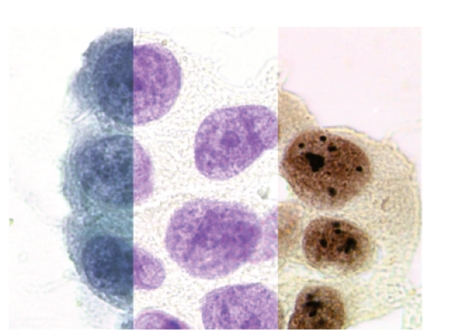
Illustration of three sequential stainings of identical oral cancer cells as performed in multimodal cell analysis [27]: Papanicolaou, Feulgen for DNA analysis, and silver nitrate for AgNOR analysis. Black dots represent AgNORs.

Another auxiliary method that allows assessment of potential malignancy of dysplastic or regenerating cells is AgNOR analysis. AgNORs represent silver-stainable nucleolar organizer regions (Figure 5). Their number and size are related to protein synthesis. Remmerbach et al. [13, 23] showed that counting the number of silver nitrate-stained nucleolar organizer regions (AgNORs) in about 100 atypical squamous cells allows 100% sensitivity and specificity of oral cancer detection on brush biopsies. 

Both methods, DNA-ICM and AgNOR analysis, may even be performed sequentially on identical cells (Figure 5). This type of multimodal cell analysis is especially useful if only few atypical cells are available [23]. Thus, AgNOR analysis can be combined with DNA-ICM if the latter does not yield an unequivocal result.

## 7. Role in Therapy

Treatment method of choice in patients with squamous cell carcinomas of the head and neck area is still surgical resection of the tumor and dissection of the regional lymph nodes. Although options for repair and restoration (e.g., free flaps) of skin and bone defects after primary surgery have improved significantly in the last decades, patients with squamous cell carcinomas of the oral cavity have only a fair prognosis with an overall 5-year survival rate of about 45% [1]. This figure has not substantially improved during the past 30 years [2, 28, 29]. Locoregional failure after surgery or even after combined surgery and radiotherapy is the main cause of death in patients with squamous cell carcinomas of the mandibular region and the maxilla. The main principle in tumor surgery is the effort to achieve tumor-free resection margins. 

Several authors have evaluated the relationship between locoregional recurrence of the tumor and the status of the resection margins [30, 31]. The prevalence of tumoral infiltration at the resection margins varies from 3.5% to 60% [30] and is usually an indicator for additional excision, postoperative irradiation, and strict followup [32]. The recurrence rate in patients with positive surgical margins treated only by surgery ranges from 36% [31] to 64% [30], and when postoperative radiotherapy is used, the recurrence rate decreases to 31% [30]. Due to the fact that it can be difficult to distinguish between squamous cell carcinomas and other lesions of the oral mucosa using only haematoxylin and eosin-stained sections [33] the resection margins are routinely examined by immunohistology. Nevertheless, the histological diagnoses of oral mucosa lesions fail sometimes [34, 35]. These days, an alternative method for the examination of oral lesions is exfoliative cytology. It is based on the technique of Papanicolaou, which is accepted worldwide, as a successful method in order to screen for epithelial dysplasias in situ or invasive carcinomas of the uteri cervix. Moreover, DNA image cytometry has been introduced for diagnosis of malignant transformation of squamous epithelial cells as an adjuvant tool to the cytological examination [20, 36]. This is used to detect the cytometric equivalent of chromosomal or DNA aneuploidy [37], which is accepted as a marker for the neoplastic transformation of cells. DNA image cytometry has been introduced as an adjuvant tool for the detection of these cell transformations in oral mucosa [20, 36]. The detection of DNA aneuploidy has been described as a diagnostic aid for the identification of prospective malignancy in various organs for example in dysplasias of the uterine cervix [38], suspicious cystic lesions of the neck, [39] or bile duct brushings [40]. The positive predictive value of DNA aneuploidy for the subsequent deletion of histologically confirmed cancer was 100% in cells of these tissues. In another study, the additional value of DNA image cytometry regarding the occurrence of a locoregional relapse was assessed [27]. In this study adjuvant use of DNA image cytometry showed a high positive predictive value of 87.5% with respect to the local recurrence of head and neck squamous cell carcinomas. Recently, Brandizzi and coworkers reported a ploidy analysis in oral squamous cell carcinomas using methodologic adjustments to improve the accuracy of the measurements of aggressiveness of prognostic value. Several indices of aggressiveness were analyzed in relation to the clinical-pathologic data and evolution of the patients. Two indices had a prognostic value of the degree of aggressiveness of oral SCC [41].

Taking into account that the diagnosis of tumor infiltration in the resection margins has often serious consequences (followup resection and/or postoperative irradiation), the presence of aneuploid cells could also change the treatment. However, it is unclear if these aneuploid cells cause the locoregional tumor relapse. Unfortunately, up to date no studies exist which confirm this. Thus, it has to be investigated in a consecutive clinical trial, whether the additional or modified treatment leads to a longer relapse-free period.

In conclusion, the additional use of DNA image cytometry is a reasonable tool for the assessment of the resection margins of SCCs. DNA image cytometry could help to find the appropriate treatment option for the patients and thus might improve their prognosis.

## 8. Followup Care

Local recurrences of oral cancer after operation are frequent events, more often following R 1/2—but even after R0—resections [27]. Exfoliative cytology allows the non-invasive evaluation of macroscopically suspicious mucosal lesions that may appear after resection. As brush biopsies are better tolerated by patients than scalpel biopsies, they may be performed more often. Thus, recurrences may be identified earlier.

## 9. Conclusion

DNA image cytometry has tremendous impact on early diagnosis and therapeutical considerations in head and neck squamous cell cancer. While oral lesions that macroscopically are urgently suspicious for cancer shall be submitted to scalpel biopsy and histologic evaluation, the majority of facultatively precancerous lesions, such as leuko- and erythroplakias or even persistent lichen planus lesions, may be assessed by brush biopsy and cytology. As this non-invasive procedure is well tolerated by patients, more lesions may be screened and thus more oral cancers may be found in early, curable stages. Oral brush biopsies can easily be performed by dentists, dental surgeons, and general practitioners. While sensitivity of exfoliative cytology alone is about 4% less than bioptic histology, the combination of the latter with DNA image cytometry reaches the same diagnostic accuracy as the former. As clonal chromosomal aneuploidy and DNA aneuploidy mostly precede cytological and histological evidence of malignancy in the squamous epithelium, its detection allows the diagnosis of oral squamous cell carcinomas up to two years earlier. Moreover, the additional use of DNA image cytometry is a reasonable tool for the assessment of the resection margins of squamous cell carcinomas. DNA image cytometry could help to find the appropriate treatment option for the patients and thus might improve their prognosis.

Finally, diagnostic DNA image cytometry is an accurate method and has internationally been standardized. Actually, it is paid by the German health insurances.
